# Source apportionment of micronutrients in the diets of Kilimanjaro,Tanzania and Counties of Western Kenya

**DOI:** 10.1038/s41598-019-51075-2

**Published:** 2019-10-08

**Authors:** Michael J. Watts, Daniel R. S. Middleton, Andrew L. Marriott, Olivier S. Humphrey, Elliott M. Hamilton, Amanda Gardner, Martin Smith, Valerie A. McCormack, Diana Menya, Michael O. Munishi, Blandina T. Mmbaga, Odipo Osano

**Affiliations:** 1 Inorganic Geochemistry, Centre for Environmental Geochemistry, British Geological Survey, Nottingham, UK; 2 Section of Environment and Radiation, International Agency for Research on Cancer, Lyon, France; 3 School of Public Health, Moi University, Eldoret, Kenya; 4 Kilimanjaro Clinical Research Institute, Moshi, Tanzania; 5 School of Environmental Sciences, University of Eldoret, Eldoret, Kenya

**Keywords:** Risk factors, Biogeochemistry

## Abstract

Soil, water and food supply composition data have been combined to primarily estimate micronutrient intakes and subsequent risk of deficiencies in each of the regions studied by generating new data to supplement and update existing food balance sheets. These data capture environmental influences, such as soil chemistry and the drinking water sources to provide spatially resolved crop and drinking water composition data, where combined information is currently limited, to better inform intervention strategies to target micronutrient deficiencies. Approximately 1500 crop samples were analysed, representing 86 food items across 50 sites in Tanzania in 2013 and >230 sites in Western Kenya between 2014 and 2018. Samples were analysed by ICP-MS for 58 elements, with this paper focussing on calcium (Ca), copper (Cu), iron (Fe), magnesium (Mg), selenium (Se), iodine (I), zinc (Zn) and molybdenum (Mo). In general, micronutrient supply from food groups was higher from Kilimanjaro,Tanzania than Counties in Western Kenya, albeit from a smaller sample. For both countries leafy vegetable and vegetable food groups consistently contained higher median micronutrient concentrations compared to other plant based food groups. Overall, calculated deficiency rates were <1% for Cu and Mo and close to or >90% for Ca, Zn and I in both countries. For Mg, a slightly lower risk of deficiency was calculated for Tanzania at 0 to 1% across simplified soil classifications and for female/males, compared to 3 to 20% for Kenya. A significant difference was observed for Se, where a 3 to 28% risk of deficiency was calculated for Tanzania compared to 93 to 100% in Kenya. Overall, 11 soil predictor variables, including pH and organic matter accounted for a small proportion of the variance in the elemental concentration of food. Tanzanian drinking water presented several opportunities for delivering greater than 10% of the estimated average requirement (EAR) for micronutrients. For example, 1 to 56% of the EAR for I and up to 10% for Se or 37% for Zn could be contributed via drinking water.

## Introduction

Mineral micronutrient deficiencies (MND) are often referred to as ‘hidden hunger’, which can result in severe health problems, and are widespread with up to two billion people worldwide at risk of iodine (I), iron (Fe), zinc (Zn), selenium (Se) calcium (Ca) and magnesium (Mg) deficiencies^1–4^. These MNDs have a considerable socio-economic burden on developing countries, particularly in sub-Saharan Africa, for populations who have limited dietary diversity and depend on locally sourced food. Joy *et al*.^5^ estimated risk of MNDs for the region to be 54, 40, 28, 19 and 5% for Ca, Zn, Se, I and Fe, respectively. Beal *et al*.^6^, reported a decrease for MNDs in most regions over the last 50 years using a Prevalence of Inadequate Micronutrient Intake Index (PMII), except for sub-Saharan Africa where it only started to decrease from 1990. The significance of MND in a developmental context was highlighted by Muthayya *et al*.^7^ who reported MND to be responsible for 1.5–12% of total Disability Adjusted Life Years (DALYS) for sub-Saharan Africa. Whilst the MNs mentioned have a vast body of evidence describing the implications for MNDs, molybdenum (Mo) does not have the same degree of interest from the literature, although it is an essential MN as a cofactor for key enzymes, including sulphite oxidase, which is required for the metabolism of sulphur amino acids^8^. The authors are not aware of reports in the literature for widespread Mo dietary deficiency, although data for Mo daily intake requirements for humans are given in food balance sheets prepared by the Food and Agriculture Organisation and therefore warrants further investigation alongside the commonly reported MNs (FAO)^9^.

Many African soils are deficient in soil-MN content or have limitations on soil-to-plant MN transfer which can impact on crop productivity and their nutritional quality. Deficiency of soil-MN in their inorganic form can largely be attributed to their parent material, whilst poor soil retention or soil-to-crop transfer is influenced by soil pH, organic matter content, redox conditions (e.g. Fe oxides, aluminium (Al)), clay/mineralogy content and mycorrhizae^10–15^. For example, Se deficiency is likely among populations on low-pH soils, but not on calcareous soils of pH greater than 6.5^13,16^. Siyame *et al*.^17^ reported Fe and haemoglobin concentrations to be higher in people residing on low pH soils in Malawi. Dickinson *et al*.^18,19^ also reported higher blood Fe and haemoglobin, but lower Se concentrations in a cohort of pregnant women living in southern Malawi on low pH soils. In Central Anatolia, Cakmak *et al*.^20,21^ reported Zn deficiency in what is a major wheat production region, where pH ranged from 7.5 to 8.1 along with low organic matter (<2%) and calcium carbonate, factors known to reduce Zn bioavailability in soils, with the exception of organic matter^22,23^. A very weak correlation was reported in Malawi for paired plant-soil I concentrations between key soil parameters or soil type and plant-I concentration^24^. The link between environmental-I and human-I status will most likely be influenced via consumption from animal food sources. In particular, dairy products or freshwater/seafood, resulting in considerable variation in I-sufficiency or deficiency, particularly when considered alongside the use of iodised salt. Groundwater drinking water supplies also have the potential to contribute significantly to I intake^24–26^.

In addition, ‘anti-nutrients’, such as phytic acid essential for growth production in seed grains, inhibits the human uptake of Zn, Fe, Mg and Ca^6,27,28^; goitrogens inhibit I uptake^29^, or infections that impair gut absorption^30^ can all exacerbate MND’s. For example, Siyame *et al*.^15^ reported low Zn plasma concentrations in Malawi were likely due to low Zn intakes combined with high Phytate: Zn ratios, a widely accepted predictor of Zn bioavailability^27^. Cyanogenic glucosides, glucosinolates and thiocyanate in common vegetables affect thyroid hormone synthesis via inhibition of iodide uptake and/or activity of thyroid peroxidase can result in the persistence of endemic goitre, even after supplementation by iodised salt^31^. Intestinal infections can reduce the availability of MN’s with sometimes increased risk of reinfection through geophagic practices^30^ which are often used to supplement mineral intake, but in themselves may not, for example, supply bioavailable Fe or reduce adsorption capacity of Fe, Zn, Mg, Ca and Se^32,33^.

Health status associated with MND are often identified through routine biomonitoring (e.g. urine for I or blood plasma for Se), which can be expensive and require rigorous quality assurance controls, are logistically challenging and expensive for large-scale surveys^34^. Biomarkers may not be adequately sensitive in determining sufficiency/deficiency status, as for Zn^35^ and can be constrained by the availability of appropriate laboratory techniques. However, biomonitoring programmes have considerable potential for a snapshot of population MN status as methodologies improve in sensitivity and robustness^36^, as already demonstrated for urinary-I^1,25,37^, Zn and Se^34,38^.

Direct assessment of health outcomes, such as stunting (Zn)^39^ and goitre (I)^40^ can be useful for assessing the prevalence of MND relative to dietary MND, but can be skewed by multiple causes of the same health condition or disease^41^. Adequacy of specific MN intake for small populations can also be quantified using composite diet analysis or dietary recall with composition data^13,17,19^. Household surveys or Food and Agriculture Organisation (FAO) Food Balance Sheets (FBS) are utilised for larger populations to derive consumption data for MNs^42^ to better inform public health policies in addressing deficiencies. However, the FBS is dependent on locally-relevant food composition data which may be lacking in low-income countries where there is a lack of infrastructure to support the analyses of all MNs in food groups, some of which are measured at parts-per-billion concentrations (e.g. Se, I) and often requires sophisticated equipment such as ICP-MS^43^. Country specific food composition data are often lacking and instead utilise a small number of datasets often from high income countries^44^ or from analyses that are decades old^45^. Considerations from other sources, such as Fe from cooking pots or dust on vegetables are possible, but little evidence is available to demonstrate the contribution to dietary intakes^35^. Newly derived food and soil analyses can update estimates of the amount of each element consumed, better inform dietary recall or FBS data that avoid incorrect assumptions and is meaningful for subsequent intervention strategies.

Along with improved information on soil chemistry, crop chemistry data can reveal regional differences influenced by changes in soil type, pH, geology and geographical features, to which bespoke interventions can be devised to ensure MN intake remains within the narrow margin between sufficiency and excess^46,47^ for both human or animals and for crop productivity^48^. For example, Joy *et al*.^14,27^ reported that Ca, Cu, Fe, Mg, Se and Zn concentrations were higher in maize grain and Ca, Cu, Fe and Se higher in leafy vegetables from calcareous (pH > 6.5) rather than non-calcareous soils. Specific soil and food composition data for estimates of dietary mineral supplies will be particularly powerful where subsistence farming is dominant (e.g. food grown locally with limited diversity of soil type/pH) and will better inform estimates of dietary diversity, especially in sub-Saharan Africa^49,50^. The methods described for determining the incidence of MND’s allow for the planning of intervention methods and close monitoring of programmes targeting the United Nations strategic development goals (SDG 2: zero hunger; SDG 3: good health & well-being). For example, interventions such as iodisation of salt^51,52^, biofortification of crops with Se, Zn^27,53–57^, agronomic practices^58^ or direct supplementation (e.g. Fe)^59^.

This study aimed to undertake a survey of soil, drinking water and staple crops in Kilimanjaro District-Tanzania and several districts in Western Kenya with the following objectives: (1) to generate crop composition data to provide new estimates of dietary MN supplies and subsequently the risk of MND by incorporating FBS supply data; (2) investigate influence of soil chemistry on crop MN composition and implication for MND rates; (3) consider drinking water as a contributory factor to dietary intake of MN’s.

## Results

All data are reported as median (Q1, Q3) in Supplementary Tables, unless stated otherwise. Crop data are presented as dry weight (DW; n = 1496). Soil (n = 282) and plant samples were collected across simplified soil categories with calcareous and non-calcareous characteristics^27^. For ease of description, these regions will be referred to simply as ‘Kenya’ and ‘Tanzania’.

### Soil composition

**Figure 1:**
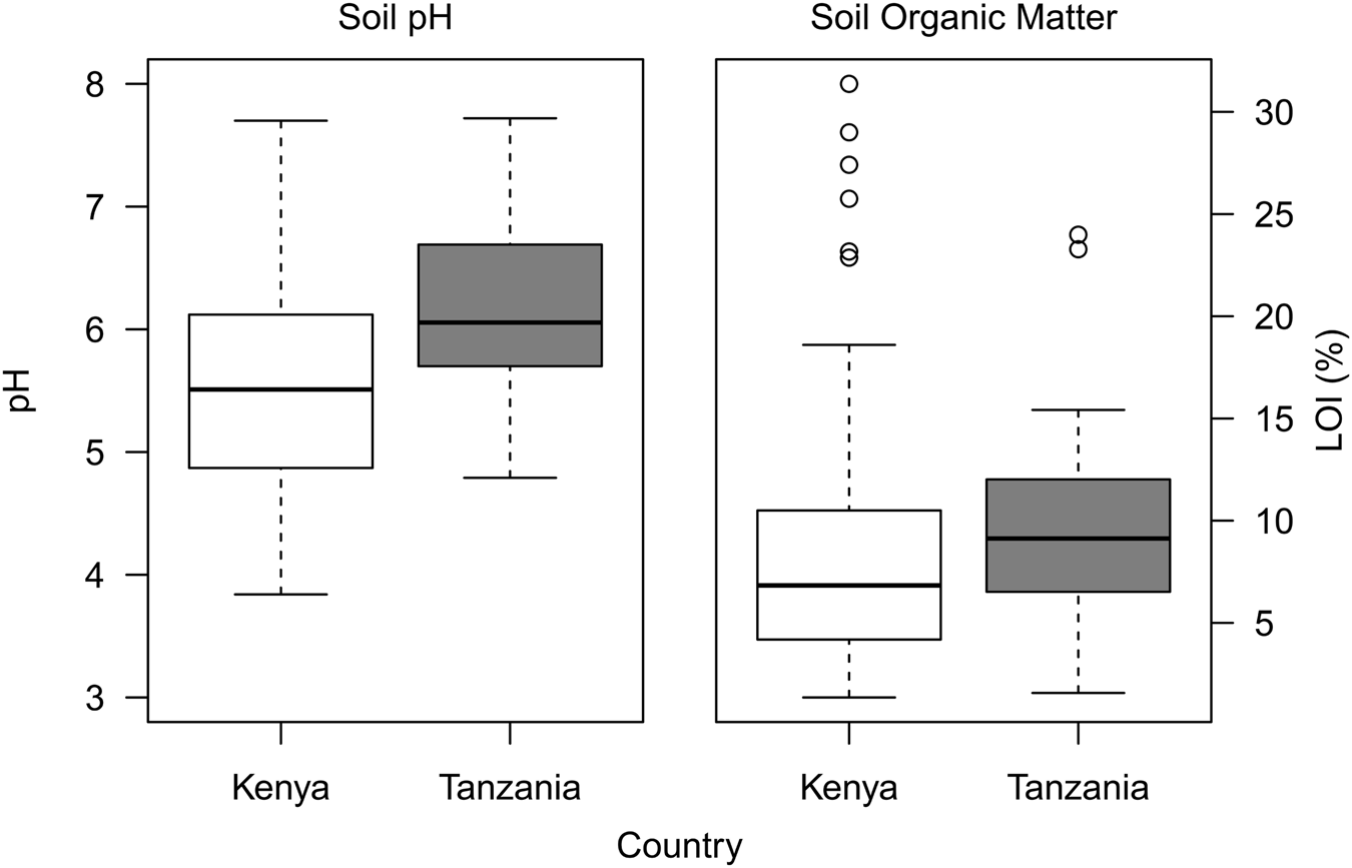
Illustration of range of soil pH and %LOI in Tanzanian and Kenyan soils.

Soil chemical and physical analyses are presented fully in Supplementary Table 1 with site metadata, uncertainty on measurements and limits of detection for analyses, whilst soil data has been summarised in Table 1. Median Ca concentrations for calcareous soils were 18,117 and 8,269 mg kg^−1^, with a median pH of 7.0 and 7.1 in Tanzania and Kenya, respectively. In comparison, the median Ca concentration in non-calcareous soils was 7,643 and 2,776 mg kg^−1^ and pH of 5.8 and 5.3 in Tanzania and Kenya, respectively. Soils were classified as calcareous where pH >6.5, although several soils considered to be non-calcareous had unexpectedly high Ca concentrations (Ca >20,000 mg kg^−1^) as similarly reported in Joy *et al*.^14^ for Malawian soils. Overall, the higher pH and Ca concentrations of calcareous soils compared to non-calcareous soils supported this approach. Median MN data for soil samples is summarised in Table 1, whilst broader information for soil location/chemical parameters and descriptive statistics are reported fully for all samples in Supplementary Tables 1 and 2, respectively. Loss-on-ignition (%) as a measure of organic matter presented a median value of 9.4 (2.3–24.0) and 5.6 (1.6–13.5) for non-calcareous and calcareous soils, respectively for Tanzania compared to 6.0 (1.4–72.2) and 6.2 (2.6–26.0) for non-calcareous and calcareous soils, respectively for Kenya. Figure 1 illustrates the range of pH and LOI values across the soils, which the median values do not convey completely, particularly for the range of LOI values.

**Table 1:** Median micronutrient concentrations for soil samples, as mg kg^−1^ (Tanzania, n = 50; Kenya n = 232).

Soil type	n	pH	%LOI	Ca	Cu	Fe	Mg	Zn	Se	I	Mo
**Tanzania**											
Non-Calc.	36	5.8	9.4	7643	111	118845	4921	191	0.9	18.0	4.2
Calcareous	14	7.0	5.6	18117	48	68098	6438	138	0.4	10.5	1.8
**Kenya**											
Non-Calc.	197	5.3	6.9	2777	19	45756	2006	110	0.6	10.0	3.1
Calcareous	35	7.1	6.2	8269	24	52639	3506	128	0.4	5.7	2.1

### Plant composition

Plant composition data and location information, along with paired data for soil are presented in Supplementary Table 3 and the number of samples for each food item in Supplementary Table 4. The supply data for each food type based on food supply estimates of intake for Tanzania and Kenya^60,61^, along with a correction for moisture content for fresh weight to dry weight are shown in Supplementary Table 5. Summary data for key food groups are presented in Table 2 for specific MN – descriptive statistics for each food item and food group are summarised in Supplementary Tables 6, 7, 8 and 9. Key soil predictor variables were used to estimate the percentage of variance in crop element concentrations explained by soil predictor variables via a multiple linear regression model using log-transformed element concentrations (Table 3).

**Table 2:** Summary of median concentrations in mg kg^−1^ (dry weight) for food groups in both countries, with descriptive statistics summarised in full in Supplementary Tables 7 and 8 for Tanzania and Kenya, respectively.

Food group	Tanzania							
	Ca	Cu	Fe	Mg	Zn	Se	I	Mo
Leafy Veg.	35,614	31.0	534	8,985	54	0.094	0.242	2.1
Vegetables	6,649	210.4	698	2,723	36	0.071	0.635	1.7
Root/tuber	259	6.6	19	1,122	14	0.013	0.005	0.3
Fruit	1,171	7.6	32	1,585	18	0.062	0.007	0.2
Grain	243	2.9	146	1,169	15	0.012	0.044	1.2
Pulses	1,038	8.6	123	1,879	32	0.143	0.008	2.8
Maize	52	2.9	26	1,188	17	0.141	0.004	0.4
Seeds	1,645	14.5	125	2,891	38	0.303	0.014	0.3
	**Kenya**							
	**Ca**	**Cu**	**Fe**	**Mg**	**Zn**	**Se**	**I**	**Mo**
Leafy Veg.	14,983	43.2	326	5,489	49	0.055	0.087	0.8
Vegetables	3,962	39.5	119	2,714	31	0.030	0.004	0.4
Root/tuber	1,119	14.3	44	1,380	18	0.021	0.010	0.1
Fruit	1,062	15.9	29	1,579	17	0.017	0.007	0.2
Grain	510	12.2	105	1,445	24	0.042	0.020	0.3
Pulses	2,151	15.0	141	2,331	38	0.041	0.010	0.8
Maize	56	3.4	24	941	20	0.030	0.004	0.2
Seeds	1,054	22.9	156	3,665	56	0.059	0.004	0.9
Nuts	592	23.9	54	2,296	38	0.094	0.004	0.5

**Table 3:** Percentage of variance in crop element concentrations explained by soil predictor variables.

Predictor	Ca	Cu	Fe	Mg	Se	Zn	I	Mo
***Maize***								
Soil element ▲	0.20 (−)	0.16 (−)	1.02	2.53	2.37*	1.31	0.30 (−)	0.92
Soil pH	0.68	0.29 (−)	0.40	0.25	5.04	2.16*	0.81	4.92
Soil OM	0.14 (−)	2.22**	0.16 (−)	0.75	5.83* (−)	0.19	0.48	1.89*(−)
Soil Fe	1.82**(−)	0.35	—	2.85	0.99* (−)	1.11	0.73	0.80
Soil Mn	0.46	0.87 (−)	1.99	1.01 (−)	0.83	3.89	2.38 (−)	0.54 (−)
Soil Al	0.37	0.08 (−)	0.33	1.99	0.33	5.52**(−)	0.66	0.77 (−)
Soil Ca	—	0.58 (−)	0.35 (−)	1.46 (−)	4.48*	1.38*(−)	1.67 (−)	5.92**
Soil Mg	0.17	2.10	0.27 (−)	—	1.56 (−)	0.63	1.90 (−)	2.32 (−)
Soil K	0.17 (−)	0.08	0.18 (−)	0.39 (−)	5.86** (−)	0.80	0.16	1.25*(−)
Soil P	0.99	0.29 (−)	0.26	6.86**	0.86 (−)	1.24 (−)	1.20 (−)	0.84 (−)
Soil S	0.34 (−)	0.40 (−)	0.52 (−)	0.47 (−)	0.96* (−)	1.47	2.76 (−)	0.66 (−)
Total variance ■	5.35	7.44	5.48	18.56	29.09	19.7	13.05	20.83
***Pulses***								
Soil element ▲	0.75 (−)	0.27	0.32	0.75	0.54	7.51**	0.46 (−)	0.98
Soil pH	0.67 (−)	0.97 (−)	0.16	0.35 (−)	4.36	0.88 (−)	1.17	5.08
Soil OM	0.27	0.99 (−)	0.08	0.48 (−)	4.70** (−)	0.60	2.58	0.93*(−)
Soil Fe	1.83 (−)	0.53 (−)	—	1.46 (−)	1.05 (−)	0.45	0.42 (−)	1.41 (−)
Soil Mn	1.67 (−)	0.28	0.20 (−)	0.97	2.13*	0.53 (−)	0.13	0.60
Soil Al	0.46	0.16 (−)	0.78	0.37	0.38	0.81 (−)	0.50 (−)	4.39**(−)
Soil Ca	—	0.94 (−)	0.52	1.18 (−)	2.11	0.64 (−)	3.08 (−)	3.37
Soil Mg	0.86 (−)	0.38	1.11 (−)	—	1.50	0.64 (−)	1.17	3.72*
Soil K	0.86	0.82	1.10 (−)	0.13	3.95* (−)	0.19 (−)	1.45 (−)	2.19*(−)
Soil P	0.96 (−)	0.42	0.74 (−)	0.71	0.63 (−)	3.41*(−)	0.36	2.05
Soil S	5.30**(−)	1.30 (−)	0.12 (−)	2.88 (−)	0.20 (−)	0.10 (−)	0.79 (−)	1.21
Total variance ■	13.64	7.07	5.13	9.28	21.54	15.74	12.12	25.94
***Green leafy vegetables***								
Soil element ▲	0.40	0.74*(−)	0.22	0.80**	5.26**	1.86*	1.98	1.93*
Soil pH	0.70	0.44	0.24	0.30	1.09	0.77	1.31	2.85
Soil OM	0.69 (−)	0.22 (−)	0.92**(−)	0.08 (−)	3.96* (−)	1.97**	0.22	0.47*(−)
Soil Fe	0.54 (−)	2.71**	—	0.21 (−)	1.85	0.37 (−)	0.51	1.19*(−)
Soil Mn	0.48	2.64	0.42	0.10 (−)	3.17	0.34 (−)	0.37 (−)	0.57 (−)
Soil Al	0.67 (−)	1.29*(−)	0.13 (−)	0.06	1.16* (−)	1.13	0.44	0.55 (−)
Soil Ca	—	0.49	0.09	0.51 (−)	0.87*	1.20*(−)	0.65	3.36**
Soil Mg	0.41	0.26 (−)	0.10	—	0.54 (−)	0.46	0.90	1.05
Soil K	0.16 (−)	0.13 (−)	0.47 (−)	0.52 (−)	0.48 (−)	0.17	1.32 (−)	0.78*(−)
Soil P	0.60 (−)	0.37 (−)	0.59 (−)	0.62	0.89	0.27 (−)	0.42 (−)	1.72*
Soil S	0.76	0.81	0.91*	0.39	1.54	0.33	2.14 (−)	0.72 (−)
Total variance ■	5.41	10.11	4.1	3.59	20.8	8.87	10.27	15.17
***Fruit***								
Soil element ▲	0.67 (−)	1.05	0.29	0.43	1.91*	0.97	1.07	0.59
Soil pH	0.20	1.55 (−)	0.04 (−)	0.66	1.81	0.23	2.36 (−)	2.94**
Soil OM	6.63**(−)	0.52	0.68 (−)	1.26 (−)	3.83** (−)	0.19	1.76 (−)	0.86 (−)
Soil Fe	0.95 (−)	0.62	—	0.45 (−)	0.39	0.61	4.38**	0.44
Soil Mn	0.99	0.21	0.18	0.35	0.96	0.47 (−)	0.27 (−)	0.19 (−)
Soil Al	0.70 (−)	0.33 (−)	0.59 (−)	1.21 (−)	0.76 (−)	1.44 (−)	0.59 (−)	1.57 (−)
Soil Ca	—	0.89	0.20 (−)	1.31** (−)	1.04	0.89 (−)	1.45	0.62 (−)
Soil Mg	1.20*	0.74 (−)	0.12	—	0.54	0.74	1.43*(−)	0.46
Soil K	1.85	1.26	0.85	0.16	0.10	1.62	0.89	0.10
Soil P	1.81 (−)	4.60**	0.26	0.44	0.67	2.22 (−)	0.32	0.18
Soil S	0.55 (−)	0.08 (−)	0.86 (−)	0.17 (−)	0.15 (−)	0.08 (−)	1.96	0.16
Total variance ■	15.56	11.84 (−)	4.06	6.44	12.15	9.46	16.49	8.1
***Roots & tubers***								
Soil element ▲	1.64	2.84 (−)	1.30	0.20 (−)	5.54**	3.42	1.80	1.74*
Soil pH	1.12 (−)	0.24 (−)	0.16 (−)	1.91 (−)	0.97 (−)	0.50 (−)	0.40	2.82 (−)
Soil OM	4.14 (−)	0.30 (−)	0.49 (−)	0.46 (−)	4.40* (−)	0.54 (−)	0.25	0.87 (−)
Soil Fe	1.86 (−)	0.74 (−)	—	0.46 (−)	1.20 (−)	0.81 (−)	0.60	0.60 (−)
Soil Mn	1.60	2.68	2.63	1.17	4.64	1.83	7.09	0.67 (−)
Soil Al	4.62 (−)	0.44 (−)	0.46 (−)	0.22 (−)	1.30 (−)	0.63 (−)	2.88 (−)	1.05 (−)
Soil Ca	—	0.93	0.18	0.39	2.56	0.49	0.35	9.18**
Soil Mg	0.57 (−)	0.67	0.23 (−)	—	0.69 (−)	1.19 (−)	0.90 (−)	2.00 (−)
Soil K	0.80 (−)	1.65 (−)	0.13	0.38	1.35 (−)	0.32 (−)	0.54 (−)	1.84*(−)
Soil P	5.24 (−)	1.02 (−)	1.04 (−)	0.16	2.98 (−)	0.68 (−)	2.93 (−)	0.64 (−)
Soil S	4.70**	3.34	7.14**	8.72**	1.41	9.52**	0.34 (−)	1.25
Total variance ■	26.31	14.86	13.75	14.06	27.05	19.94	18.09	22.66

Estimates are from multiple linear regression models using log-transformed element concentrations. Significant (p < 0.05) predictors are shown as (*), dominant significant predictors are shown as (**). Directions of association are positive unless shown otherwise (−).▲Concentration of response variable element in soils; ■Total variance explained by modelled predictors.

The percentage of the population at risk of MN deficiency was calculated in Supplementary Tables 10 and 11, with full workings shown. Summarised deficiency rates are shown in Table 4 across selected MNs for: both male and female to account for differing EARs, by soil type whereby food sources originated from either calcareous or non-calcareous soils, and for each country.

**Table 4:** Summary of % population at risk of micronutrient deficiency according to the Estimated Average Requirement (EAR) for female (F) and male (m) for both Tanzania and Kenya, for diets originating from either non-calcareous or calcareous soils.

% Deficiency	Gender	Ca	Cu	Fe	Mg	Se	Zn	I	Mo
**Tanzania**									
Non-calcareous	F	95	0	100	0	8	86	100	0
	M	95	0	20	1	28	100	100	0
Calcareous	F	89	0	99	0	3	90	100	0
	M	89	0	7	1	12	100	100	0
Published data	F	100	1	100	8	55	99	100	0
	M	100	1	100	18	92	100	100	0
**Kenya**									
Non-calcareous	F	100	0	100	3	93	85	100	0
	M	100	0	100	6	100	100	100	0
Calcareous	F	100	0	100	9	100	98	100	0
	M	100	0	100	20	100	100	100	0
Published data	F	100	3	100	11	73	100	100	0
	M	100	3	100	23	98	100	100	0

### Drinking water concentrations

Drinking water composition data, as well as location, source and usage data is shown in full in Supplementary Table 12 and summarised in Table 5 for selected MNs. Descriptive statistics for all measured determinands are presented by source of drinking water in Supplementary Tables 13 and 14, for Tanzania and Kenya, respectively and descriptive statistics for both countries in Supplementary Table 15. Table 6 summarises the potential percentage contribution that drinking water could make to the EAR for each MN, based on assumed daily consumption of 1.7 L per day^6^.

**Table 5:** Summary of median drinking water values for MNs by source for each country – complete data reported in Supplementary Tables 13 and 14.

Source	n	pH	NPOC (mg L^−1^)	Conductivity (µS cm^−1^)	TDS (mg L^−1^)	Ca (mg L^−1^)	Cu (µg L^−1^)	Fe (µg L^−1^)	Mg (mg L^−1^)	Zn (µg L^−1^)	Se (µg L^−1^)	I (µg L^−1^)	Mo (µg L^−1^)
**Tanzania**													
Piped	35	7.4	1.1	76	66	5.3	0.9	6.9	2.4	32	0.07	1.3	0.1
Well	2	8.3	1.2	1200	740	8.1	0.2	1.4	3.6	1	1.98	35.4	14.9
Rainwater	2	7.5	1.2	70	53	8.9	0.8	1.2	1.0	24	0.10	1.0	0.1
Spring	1	6.3	0.3	67	49	2.4	0.2	1.0	1.3	4	0.05	5.5	0.1
Borehole	1	7.6	2.7	817	598	64.5	1.2	1.0	32.6	41	0.05	49.7	1.0
Surface	2	8.0	3.0	345	274	16.6	0.7	17.8	9.0	2	0.05	22.4	1.7
**Kenya**													
Piped	35	7.4	1.7	96	55	5.8	0.7	3.9	1.6	65	0.05	4.4	0.3
Well	64	7.2	0.9	112	77	6.2	0.4	8.1	1.8	7	0.07	3.5	0.1
Rainwater	29	6.7	1.5	34	25	2.1	0.6	4.9	0.3	282	0.08	1.8	0.1
Spring	58	7.3	0.8	100	56	6.1	0.3	4.2	2.1	8	0.07	2.0	0.1
Borehole	27	7.3	0.6	152	85	9.2	0.4	2.4	2.4	13	0.09	3.9	0.1
Surface	41	7.6	2.2	119	77	6.8	0.5	27.0	2.1	8	0.08	6.5	0.3
Undefined	3	7.9	1.1	296	175	23.7	0.4	8.9	8.8	21	0.13	5.3	0.3

**Table 6:** Percentage contribution of drinking water, by source for female (F) and male (M) to the Estimated Average Requirement for selected MNs.

	Ca	Cu	Fe		Mg		Se		Zn		I	Mo
	F&M	F&M	F	M	F	M	F	M	F	M	F&M	F&M
**Tanzania**												
Piped	0.8	0.2	0.0	0.0	1.5	1.3	0.4	0.3	0.5	0.3	1	0.5
Well	1.1	0.0	0.0	0.0	2.3	2.0	10.8	8.3	0.0	0.0	40	74.5
Rainwater	1.3	0.2	0.0	0.0	0.6	0.5	0.5	0.4	0.3	0.2	1	0.5
Spring	0.3	0.0	0.0	0.0	0.8	0.7	0.3	0.2	0.1	0.0	6	0.5
Borehole	9.1	0.3	0.0	0.0	21.0	17.8	0.3	0.2	0.6	0.4	56	5.0
Surface	2.4	0.2	0.0	0.1	5.8	4.9	0.3	0.2	0.0	0.0	25	8.5
**Kenya**												
Piped	0.8	0.2	0.0	0.0	1.0	0.9	0.3	0.2	0.9	0.7	5.0	1.5
Well	0.9	0.1	0.0	0.0	1.2	0.1	0.4	0.3	0.1	0.1	4.0	0.5
Rainwater	0.3	0.1	0.0	0.0	0.2	2.9	0.4	0.3	4.1	2.9	2.0	0.5
Spring	0.9	0.1	0.0	0.0	1.4	0.1	0.4	0.3	0.1	0.1	2.3	0.5
Borehole	1.3	0.1	0.0	0.0	1.5	0.1	0.5	0.4	0.2	0.1	4.4	0.5
Surface	1.0	0.1	0.0	0.1	1.4	0.1	0.4	0.3	0.1	0.1	7.4	1.5
Undefined	3.4	0.1	0.0	0.0	5.7	0.2	0.7	0.5	0.3	0.2	6.0	1.5

## Discussion

In general, median concentrations of soil micronutrients in Table 1 are higher in Tanzanian soils compared to Kenyan soils sampled. Tanzanian median soil concentrations are significantly higher for this study around the west, south and eastern slopes of Mount Kilimanjaro for Cu, Fe and Zn compared to values reported by Mathew *et al*.^48^ on the southern slopes at 8.49, 130 and 2.82 mg kg^−1^, respectively. Kenyan median soil concentrations are also significantly higher in this study compared Jumba *et al*.^61^ for Mount Elgon toward the western reach of this study in Kenya, for example, Ca, Cu, Fe, Mg, Zn, Se and Mo were reported as 1500, 4, 300, 1600, 24, 0.1 and 1.1 mg kg^−1^. Akengu *et al*.^62^ also reported lower values for sugarcane fields in Kakamega county, towards the centre of this studies locations in Kenya at 235–891, 0.4–11, 144–636, 3–24 mg kg^−1^ for Ca, Cu, Fe and Zn, respectively. It is likely that the lower values in these comparative studies resulted from differing techniques that did not provide a ‘true’ elemental measurement, such as aqua regia for partial dissolutions, as opposed to the use of HF/HNO_3_/HClO_4_ in this study for a ‘total’ dissolution of trace and major elements.

There is no fixed pattern across the soil micronutrients when comparing non-calcareous/calcareous soils, other than Se, I Mo median concentrations are higher in non-calcareous soils for both countries. The influence of soil type on food group micronutrient composition is difficult to discern for this dataset. For example, Table 3 summarises the percentage of variance in crop element concentrations as explained by soil predictor variables for each food group for a combined Tanzania-Kenya dataset, with not all variance explained by key soil parameters (e.g. pH, OM), although for some elements there is a stronger influence from soil chemistry. The influence of soil variables, MN supply, estimated risk of deficiency and drinking water supplies are discussed in further detail for each selected micronutrient.

### Calcium

There is no overall pattern for Ca between Tanzanian and Kenyan food groups, although for both countries leafy vegetables and vegetable groups had the highest median concentrations of Ca in Table 2, from which sukuma wiki is the notable individual food item, with 29,569 and 24,271 mg kg^−1^ for Tanzania and Kenya. The median Ca concentration in maize was slightly higher at 52 and 56 mg kg^−1^ for Tanzania and Kenya compared to Malawian maize at 34 across all soil types^27^. The total variance from soil predictor variables influencing Ca crop concentrations is generally weak (5–26%), with S, P, Al accounting for nearly 15% of the 26% variance in roots/tubers. Soil pH generally had a low influence at < 1%.

Estimated Ca supplies for food items originating from calcareous/non-calcareous soils and published composition data were 914/852/225 mg capita^−1^ day^−1^ and 307/422/213 mg capita^−1^ day^−1^ for Tanzania and Kenya, respectively. Ca supply is notably higher for Tanzania than Kenya across all values, likely due to the much higher Ca content of the leafy vegetable group. Supply values measured for both soil types are much higher compared to the use of published composition data, particularly for Tanzania. Both countries are still deficient in Ca supply compared to the EAR of 1200 mg capita^−1^ day^−1^, with the risk of deficiency ranging from 89 to 95% for male and female in Tanzania and 100% risk of Ca deficiency in Kenya, comparable to Joy *et al*.^27^ observation of >97% for Malawi, yet much higher when compared to a wider African estimation of 54%^5^. The low Ca supply for Kenya is comparable to measured estimates for Mali at 302 mg capita^−1^ day^−1^ ^63^ and Malawi at 430, 368 and 367 mg capita^−1^ day^−1^ for calcareous, non-calcareous soils and published data^27^.

Drinking water has the potential to contribute to the daily intake of MNs given that at some households water sources exhibited elevated MN concentrations. For example, Ca in drinking water as summarised in Table 5 ranged from 2.4 mg L^−1^ in spring water to 64.5 mg L^−1^ in borehole water for Tanzania and from 2.1 mg L^−1^ in rainwater to 9.2 mg L^−1^ in borehole water, or 23.7 mg L^−1^ where the source was undefined for Kenya. Assuming a conservative water consumption of 1.7 L per day^6^, as shown in Table 6, borehole water in Tanzania could supply 9% of the EAR for Ca. Using median Ca concentrations from Table 4 however, suggests a low contribution to the EAR for Ca from other water sources.

### Copper

Median Cu concentrations for food groups in Table 2 were generally higher for Kenya, with the exception of the vegetable group (Tanzania - 210 mg kg^−1^). Maize was comparatively low for both countries (~3 mg kg^−1^), whilst some individual food items were relatively high. For example, moringa seeds, chilli, leek and onion from Kenya at 145, 329, 456 and 81 mg kg^−1^, compared to onion, pumpkin leaves, sweet potato and coffee beans from Tanzania at 210, 47, 58 and 79 mg kg^−1^, respectively. The total variance for the Cu content of crops attributed to soil parameters was generally <10% and approximately 15% for roots/tubers. Variance in crop-Cu content is unlikely influenced by soil type according to calcareous/non-calcareous soils.

Estimated Cu supplies for food items originating from calcareous/non-calcareous soils and published composition data were 4.3/3.7/1.6 mg capita^−1^ day^−1^ and 7.8/4.3/1.3 mg capita^−1^ day^−1^ for Tanzania and Kenya, respectively. Cu supply is notably higher for Kenya than Tanzania for both soil types, albeit higher from calcareous soils for each country and measured values significantly higher than from published composition data. The risk of deficiency for Cu is < 1% for both countries (Table 4) using an EAR of 0.7 mg capita^−1^ day^−1^. Estimates of low Cu deficiency are consistent with previous reported studies in East Africa^5,27^. In general, drinking water sources for both countries were close to or <1 µg L^−1^ of Cu, with virtually no contribution to the EAR for Cu (Table 6).

### Iron

The leafy vegetable and vegetable food groups provided the highest median Fe concentrations for both countries shown in Table 2, from which pumpkin leaves, spinach and sukuma wiki contained 165 to 1,199 mg kg^−1^. As a staple food item maize contained comparatively low concentrations of Fe at 26 and 24 mg kg^−1^ for Tanzania and Kenya, respectively. The total variance for Fe in crops attributed to soil parameters was generally very low at <5% across food groups and 14% for roots and tubers, likely due to soil contamination.

Food supply estimations for food items originating from calcareous/non-calcareous soils and published composition data were 57.2/45.1/15.1 mg capita^−1^ day^−1^ and 19.5/20.7/14.1 mg capita^−1^ day^−1^ for Tanzania and Kenya, respectively. These values are comparable to Schmidhuber *et al*.^4^ estimates of 13 to 21 mg capita^−1^ day^−1^ for Fe from a worldwide database including Kenya and Tanzania, although this study suggests higher intake values in the Kilimanjaro district. Measured supply values are comparable to Mali of 22.8 mg capita^−1^ day^−1^ ^63^. Similarly to Ca, the estimated Fe supply is notably higher than published values for both countries, yet little difference is apparent between soil types as was observed by Siyame *et al*.^17^ in Malawi. The risk of Fe deficiency does not vary particularly between soil types, but for Tanzania differs considerably for males compared to published values, with a 20 and 7% risk of Fe deficiency for each soil, compared to 100% using published values and 100% for all female estimates. The higher risk of Fe deficiency in females is likely due to the higher EAR assigned, according to an estimate of low bioavailability from food items^64^. For Kenya, all variables in Table 3 show an estimated 100% risk of Fe deficiency in Kenya, which is significantly higher than a wider African estimation of 54% by Joy *et al*.^5^ and a 9–18% estimation for women of reproducible age by Harika *et al*.^65^ from pooled national survey data for 5 Sub-Saharan African countries, including Kenya.

Iron concentrations were generally low across all drinking water sources. For example, median Fe concentrations of 1.0 µg L^−1^ for spring and borehole water to 17.8 µg L^−1^ for surface water in Tanzania and 2.4 to 27.0 µg L^−1^ for borehole and surface water, respectively, for Kenya (Table 3). Virtually no contribution to the female and male EAR for Fe can be attributed to drinking water in both countries when using median concentration values (Table 6). However, although few, some well and surface waters contained Fe at concentrations >2,500 µg L^−1^, far exceeding guideline values of 200 µg L^−1^ ^66^, with potential for significant contributions to the EAR if used as drinking water.

### Magnesium

The leafy vegetable food group also provided the highest median concentration of Mg, at 8,985 and 5,489 mg kg^−1^ for Tanzania and Kenya, respectively, with pumpkin leaves, spinach and Sukuma wiki as the highest source of Mg from 4,944 to 13,259 mg kg^−1^. Vegetables in general and seeds are a comparatively good source of Mg and maize a poor source, similarly to the majority of micronutrients for both countries. The total variance of Mg content of crops attributed to soil predictor variables was <10% for the majority of food groups, except for Maize (19%) and roots/tubers (14%), for which soil P and soil S were the greatest contributors to variance.

Estimated Mg supplies for food items originating from calcareous/non-calcareous soils and published composition data were 875/860/402 mg capita^−1^ day^−1^ and 393/507/383 mg capita^−1^ day^−1^ for Tanzania and Kenya, respectively. The risk of Mg deficiency in both male and female across soil types is therefore low at <1% in Tanzania, particularly when using published composition data (8 to 18%) and <6% for non-calcareous soils and <20% for calcareous soils in Kenya. The low risk of Mg deficiency is comparable with wider African estimates of <1%^5^. Whilst the soil predictor variables did not show a particularly strong relationship to Mg crop composition, there is a discernible difference between soil types when considering the supply and percentage risk of Mg deficiency.

Median Mg concentrations ranged from 1.0 to 32.6 µg L^−1^ for rainwater and borehole water, respectively for Tanzania. In Kenya, Mg was generally lower, ranging from 0.3 to 8.8 µg L^−1^ in rainwater and undefined drinking water sources, respectively. Borehole water has the potential to supply 21 and 18% of the female and male EAR in Tanzania, with the other water sources <6% of the EAR for Mg (Table 6). In Kenya, the majority of water sources contribute a negligible proportion of the dietary intake for the female and male EAR, although the undefined sources could be up to 6%. All water samples were below recommended thresholds of 70 µg L^−1^ ^66^.

### Zinc

Leafy vegetables exhibited the higher median Zn concentrations for both countries of the food groups, as for other micronutrients, at 54 and 49 mg kg^−1^ and maize the lowest at 17 and 20 mg kg^−1^ for Tanzania and Kenya, respectively. Seeds recorded the highest food group for Zn from Kenya at 56 mg kg^−1^. The total variance in crop Zn composition according to soil predictor variables was <10% for food groups, with the exception of leafy vegetables (20%) and roots\tubers (20%), with soil Al and S being the largest contributors to variance.

Measured Zn supplies for food items originating from calcareous/non-calcareous soils and published composition data were not dissimilar 8.9/9.2/7.6 mg capita^−1^ day^−1^ and 7.8/9.3/6.9 mg capita^−1^ day^−1^ for Tanzania and Kenya, respectively, but for both countries measured intake values were higher than for published composition data. Zinc intake in this study is at the upper range for estimates by Schmidhuber *et al*.^4^ worldwide database for Tanzania (6.2–9.3 mg capita^−1^ day^−1^), but at the lower range for Kenya (9.3–12.4 mg capita^−1^ day^−1^). Measured supply values were comparable to Mali at 6.7 mg capita^−1^ day^−1^ ^63^. The percentage risk of deficiency was calculated against an EAR assuming a low Zn bioavailability^64^. From this data, both countries are at a significant risk of Zn deficiency, generally 100%, although for females slightly lower at 85 to 100%, due to the lower female EAR. No difference in the risk of deficiency was estimated across soil type. These estimates are significantly greater than reported by Harika *et al*.^65^ at 34% risk of deficiency for Zn from pooled national data across 5 African countries, including Kenya, yet comparable to 88% of households estimated to be at risk of Zn deficiency in Malawi^27^.

Median Zn concentrations for water sources in Tanzania ranged from 1 to 41 µg L^−1^ for well and borehole sources, respectively. In Kenya, median Zn concentrations across water sources were varied, ranging from 7 to 282 µg L^−1^ in well and rainwater, respectively (Table 5). In general, Tanzanian water supplies have the potential to deliver <1% of the female and male EAR for Zn, whereas in Kenya up to 4.1 and 2.9% of the female and male EAR for Zn could be delivered via rainwater sources which varied considerably in Zn concentrations. For example, rainwater from Kenya contained a median Zn concentration of 240 µg L^−1^, likely owing to the Zn galvanised roof from which rainwater was collected. For a household with the maximum concentration of 2567 µg L^−1^, rainwater could contribute 4.4 mg of Zn per day to supply intakes, accounting for 37 or 26% of the EAR female and males, respectively. Kujinga *et al*.^67^ explored the potential for Zn fortification of drinking water for rural pre-school children in Kisumu-Kenya and reported a contribution of 36 and 31% of daily requirements for absorbable Zn in 2–3 and 4–6 year olds, respectively. Subsequently, Zn-fortified drinking water resulted in a lower prevalence of reported illnesses, such as the common cold, abdominal pain and diarrhoea.

### Selenium

Whilst maize-Se is discernibly different between calcareous/non-calcareous at 0.19 and 0.14 mg kg^−1^ in Tanzania, there is little difference for Kenya at 0.04 and 0.03 mg kg^−1^, respectively, albeit at much lower concentrations. Maize Se was higher than published data from food balance sheet data^9,60^ at 0.029 mg kg^−1^ for both countries in this study and reported data for Malawi at 0.044 and 0.015 mg kg^−1^ for calcareous/non-calcareous soils^27^. Whilst the difference in dietary Se between soil types is significant in Malawi^13,54^ owing to the domination of maize in the diet, with a supply of 328 g capita^−1^ day^−1^ (DW), any difference resulting from soil type is likely to be less significant for Tanzania and Kenya owing to their respective lower intakes of 144 and 187 g cap^−1^ day^−1^ (DW) for maize, as shown in Supplementary Table 5, resulting from a more diversified diet and less reliance on maize in the diet.

The total percentage variance in crop element concentrations explained by soil predictor variables is generally greater for Se than other elements across all food groups. For example, 29% of the variance of Se in maize is explained by soil composition, from which soil pH, OM, Ca and K make up the larger contributions to variance. Soil pH follows a similar pattern for pulses (4.4%), but less than 2% for the other food groups, whereas OM is a consistent contributor to variance across all food groups. The concentration of Se in the soil is a significant contributor to variance in the Se content of (>5%) in leafy vegetables and roots/tubers, likely due to contamination with soil/dust particles.

Estimated Se supplies for food items originating from calcareous/non-calcareous soils in this study and published data from FAO FBS sheets^9,60^ were 0.058/0.048/0.030 mg capita^−1^ day^−1^ and 0.013/0.023/0.027 mg capita^−1^ day^−1^ for Tanzania and Kenya, respectively. Whilst the Kenyan published supply data was similar to measured values, the published supply value for Tanzania was significantly lower than the measured values. The higher Se supply from calcareous soils in Tanzania was comparable to Joy *et al*.^27^ calculations for Malawi, 0.041, 0.019 and 0.030 mg capita^−1^ day^−1^ for calcareous/non-calcareous/published data. Table 5 summarises the calculated percentage risk of deficiency in comparison to the female and male EAR. Tanzania in general exhibited a much lower risk of Se deficiency for both female and male across soil types (3 to 28%) compared to Kenya, with slightly lower rates for calcareous compared to non-calcareous soils and a much lower risk of Se deficiency when using measured versus published data (55 to 92%). Overall Kenya exhibited a high risk of Se deficiency across both soil types for female and male (93 to 100%), compared to the use of published composition values (73 to 98%) and much higher than wider African estimates of 28% by Joy *et al*.^5^. Joy *et al*.^27^ also reported a comparably high risk of Se deficiency specifically for Malawi from a national survey of food items, at up to 92%. As mentioned above, the lower proportion of maize in the Tanzanian diet compared to Malawi and Kenya and greater food diversity (e.g. dairy intake) (Supplementary Table 5) is likely a major factor in the differing Se deficiency rates, but most likely is the significantly higher Se concentration in Tanzanian maize which still constitutes approximately half of the calorific intake.

Median Se concentrations from Table 5 were generally <0.1 µg L^−1^ for all drinking water sources, with the exception of well water from Tanzania at 1.98 µg L^−1^, with the potential to deliver up to 11 and 8% of the female and male EAR for Se. However, it should be noted that the majority of drinking water samples collected in Tanzania were collected from piped supplies which were treated specifically to remove the naturally content of fluoride. The well water collected from households was primarily used for washing and cooking only, due to the high total dissolved content and salinity, with drinking water purchased in canisters. All waters were far below threshold values of 20 µg L^−1^ ^66^.

### Iodine

In general crops are a poor source of iodine owing to the limited uptake of iodine via the root system^15^. Therefore, aerial deposition is a considered, albeit limited pathway for I sorption, as shown by the leafy vegetable group with the higher I measured values at 0.242 and 0.087 mg kg^−1^ for Tanzania and Kenya, respectively. In Tanzania the vegetable group provided the highest median I at 0.635 mg kg^−1^. In general, Tanzanian food groups provided a higher measured I content than Kenya. As for other micronutrients, pumpkin leaves, spinach and sukuma wiki were the notable food items, with 0.063 to 0.310 mg kg^−1^ of I for both countries. The total variance for the I content of crops according to soil predictor variables was generally <20% across all food groups, with no particularly dominant variables, other than soil Fe (4.4%) for fruit and soil Mn (7%) for root/tubers.

Measured I supplies for food items originating from calcareous/non-calcareous soils and published composition data were not dissimilar 0.029/0.017/0.030 and 0.003/0.024/0.032 mg capita^−1^ day^−1^ for Tanzania and Kenya respectively. Measured supply data from Tanzania was comparable to using published composition data, yet for Kenyan measured values were significantly lower in calcareous soils compared to published values. The risk of I deficiency in both countries is significant (100%) according to the supply calculations for both countries, although drinking water sources and in particular, iodised salt intake are likely to alleviate the severe deficiency in food supply greatly. For example, 89% of the population of Africa would be at risk of I deficiency without iodised salt supplies, reducing the risk to 19% if iodised salt consumption is included in calculations, which contributed 63% of the total I supply in Africa. More specifically, in Kenya the use of adequately iodised salt was reported in 98% of households^5^. In comparison, Harika *et al*.^65^ reported 22–55% of WRA at risk of I deficiency in 5 African countries, including Kenya, although it is not clear whether iodised salt was included in the survey data.

Median I drinking water sources (Table 3) ranged from 1.0 to 49.7 µg L^−1^ in rainwater and borehole sources in Tanzania. In Kenya, median I concentrations were generally lower ranging from 1.8 to 6.5 µg L^−1^ for rainwater and surface water, respectively. In Tanzania, borehole water has the potential to provide up to 56% of the female and male EAR, whilst for Kenya up to 7% of the EAR for I (Table 6) surface water. Farebrother *et al*.^26^ reported similarly variable I concentrations across water sources in Kenya (median 1 µg L^−1^; 0–2943 µg L^−1^), Tanzania (median 4 µg L^−1^; 0–596 µg L^−1^) and Djibouti (median 92 µg L^−1^), with the potential for excess iodine intake via surface or groundwater sources.

### Molybdenum

For Mo, the leafy vegetable and vegetable food groups were a significant source in both countries as shown in Table 2 at 2.1/1.7 mg kg^−1^ and 0.4/0.8 mg kg^−1^ for Tanzania and Kenya, respectively. Seeds and pulses were also significant in comparison to other food groups, with the median Mo concentration in pulses from Tanzania at 2.8 mg kg^−1^. From within this food group, pigeon peas were the standout food item at 9.15 mg kg^−1^. The total variance for the Mo content of crops according to soil predictor variables ranged from 8 to 26%, with soil Ca acting as a major component for variance, alongside soil pH in all food groups, suggesting a difference in food Mo composition between calcareous and non-calcareous soils.

Measured Mo supplies for food items originating from calcareous/non-calcareous soils and published composition data were 0.40/0.36/0.50 and 0.15/0.13/0.35 mg capita^−1^ day^−1^ for Tanzania and Kenya respectively. Measured Mo supply data for Tanzania was slightly lower than using published composition data, whereas measured supply data was less than half of the published values for Kenya. Molybdenum supply was more than double across both soil types in Tanzania compared to Kenya and no obvious difference from these values between soil type in either country. The risk of deficiency across soil types and for both countries was calculated as <1%. The authors are not aware of other studies reporting deficiency rates for Mo.

Median Mo concentrations were generally <1 µg L^−1^ for all drinking water sources in both countries, with the exception of well water from Tanzania, which contained a median concentration of 14.9 µg L^−1^ Mo which could contribute up to 75% if the EAR for Mo, with borehole and surface water contributing 5 and 8.5%, respectively. However, well water was reportedly rarely used for drinking as mentioned previously. In Kenya, the contribution to the EAR for Mo was generally below 1.5%. All water samples were far below threshold values of 70 µg L^−1^ ^66^.

## Conclusion

The development of localised food composition tables and updating of existing ones at a geospatial scale will improve the accuracy of estimates for dietary intake and risk of deficiency for MNs, particularly where information is limited for localised preferences for food items and assumed at a national level periodically for the study locations. These data will inform intervention strategies, including: direct supplementation, fortification of drinking water, agronomic strategies (e.g. liming, organic reincorporation), phytofortification or promotion of nutritional diversity^50,55,67–69^ to address challenges presented by the United Nations Strategic Development Goals (SDG). Opportunities exist to address SDG 2 & 3 (zero hunger, good health & well-being), SDG 6 (Clean water & sanitation) and SDG 17 (partnerships for the goals). This spatially resolved dataset will be invaluable to multidisciplinary efforts to investigate causal effects for human and animal health^70^. For example, Schaafsma (2015)^71^ identified an ecological association between estimated national level incidence rates of oesophageal cancer and predicted deficiency of dietary Se and Zn, pointing to a potential geochemical/spatial influence.

This study uses data from several sources to supplement this new dataset, each with its own limitations. For example, food supply data are aggregated at national level and do not capture community level socioeconomic factors, seasonal variation in the type of food available or food wastage^3^. Whilst the contribution of MNs to the EAR is small from drinking water, for severely deficient individuals this could still be a valuable intake route not considered in the food composition tables, particularly given that the consumption of 1.7 L per day is likely to be an underestimate for hot climates. This study has also assessed health nutritional status for iodine^25^, but planning intervention strategies to address the SDGs will benefit further from assessment of health indicators for other MNs in combination with the calculated risks of deficiency in this study.

## Methods

### Ethical approval

For the wider study, which included human biomonitoring (not presented in this paper), the study was approved by the IARC ethics committee (IEC 14–15), the Tanzanian National Institute of Medical Research (NIMR/HQ/R.8a) and, in Kenya, the Moi University Institutional Research Ethics Committee (IREC 000921). Informed consent was obtained from all householders and methods were carried in accordance with the research guidelines and regulations.

### Sample collection and analysis

**Figure 2:**
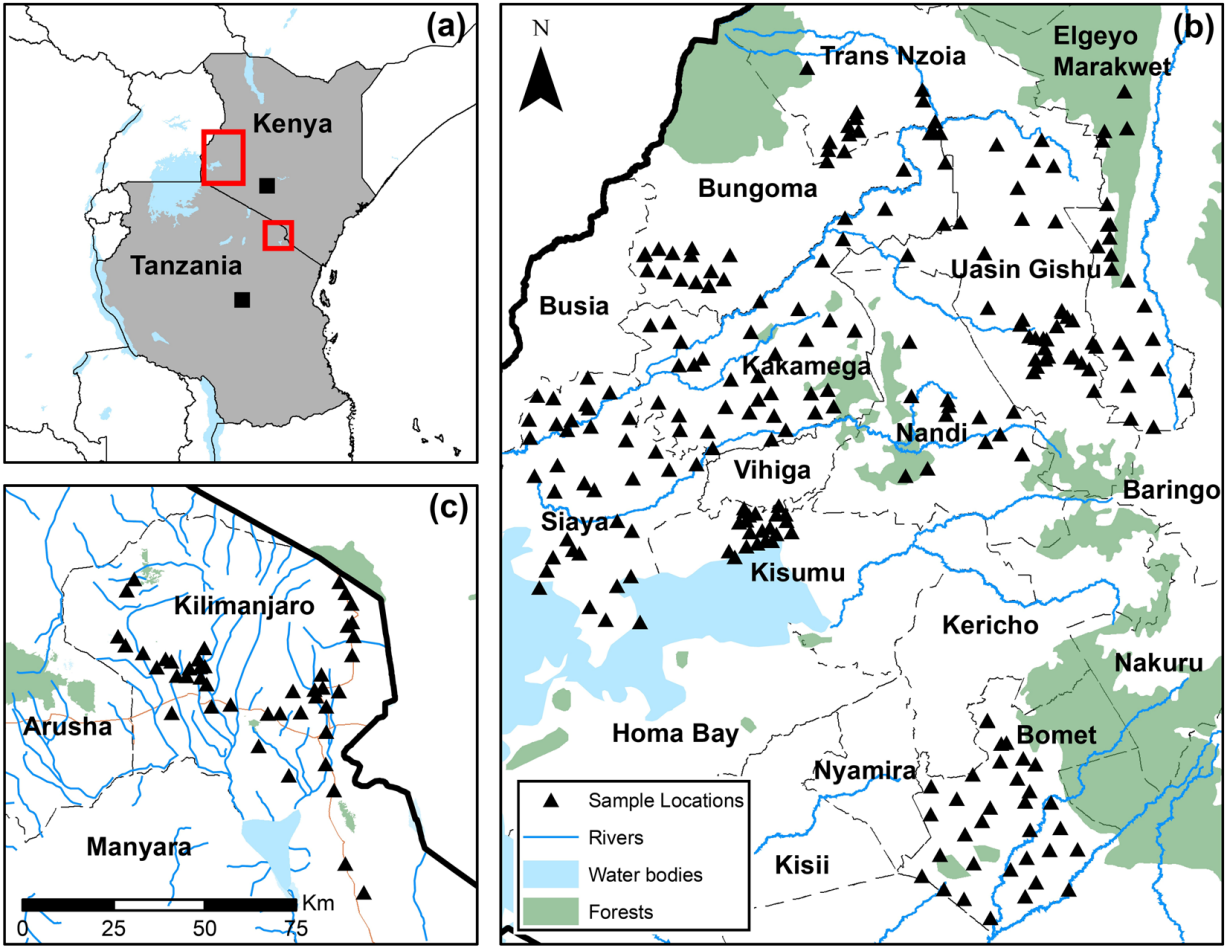
Study site locations represented by red boxes for Tanzania and Kenya in (**a**), collection locations shown in greater detail for Kenya in (**b**) and Tanzania in (**c**).

The study areas in the Kilimanjaro District of Tanzania and Counties of Bomet, Bungoma, Elgoyo Marakwet, Kakamega, Kisumu, Nandi Hills, Siaya and Uasin Gishu in Western Kenya were selected initially owing to the high oesophageal cancer rates reported, a disease for which MND has been implicated as one of the many causal factors^71,72^. Study areas will be simply referred to as ‘Tanzania’ and ‘Kenya’, which included 50 and >230 collection sites, respectively. Comparison of soil classifications will simply refer to calcareous and non-calcareous soils, as demonstrated by Joy *et al*.^27^. Plant, soil and drinking water samples were collected from household’shambas’ (produce plots) in rural locations between 2014 and 2018 at locations shown in Fig. 2.

Planning for sampling locations included engagement with each of the County Ministry of Health to obtain permissions for collections and to open a dialogue of information exchange. Public Health officers accompanied field teams within each county, which was invaluable for local knowledge and in gaining permission from each household, but also of particular importance given the additional collection of drinking water and urine samples, along with pertinent information about the location, land-use activities and potential health conditions in the vicinity.

Water and urine^25^ samples were filtered using a 0.45 µm syringe filter and stored in a coolbox until returned to the local laboratory and acidified as appropriate for ICP-MS analyses with 1%HNO3/0.5%HCl. Plant samples representing staple food groups as defined by FAO food classifications and what was commonly available at each household were brushed and washed with distilled water at each location to remove soil and dust particles and leafy vegetables air dried until return to the laboratory where they were oven dried at 40 °C. Soft fruit and vegetables were peeled, vacuum sealed and frozen at -80 °C for transport to the UK for freeze drying. Soil samples were collected at the shamba using 5 points to 15 cm depth in a 5 × 5 m grid. Soil samples were air dried, disaggregated and sieved to 2 mm. Plant samples were ground using a coffee grinder, whilst soil samples were ground to <53 µm in an agate ball mill for onward organic matter content by loss-on ignition (450 °C) and trace/major elemental analyses ICP-MS.

Soil samples (0.25 g) were dissolved as described in Joy *et al*.^27^ and Watts *et al*.^73^ for a broad suite of trace and major elements in a mixed acid solution (HF:2.5 mL/HNO_3_:2 mL/H_2_O_2_:2.5 mL) on a programmable hot block; 0.5 g of plant samples were digested in HNO_3_:10 mL/H_2_O_2_:1 mL mixed solution in a closed vessel microwave heating system (MARS Xpress, CEM). For I analyses, 0.25 g of soil was extracted using 5 mL of 5% v/v tetramethyl ammonium hydroxide (TMAH) as described in Watts *et al*.^74^, heated in a 15 mL Nalgene HDPE bottle at 70 °C in a drying oven for 3 hours and then diluted with 5 mL of deionised water followed by centrifugation at 3,000 rpm for 15 minutes, from which the supernatant was used for analysis. Plant samples required a more robust extraction for I as described in Watts *et al*.^24^; 0.25 g of plant material and 5 mL of 5% v/v TMAH were heated in a closed vessel microwave unit to 70 °C for 30 minutes, diluted with 5 mL and centrifuged as for the soils prior to analyses.

Subsequent total elemental analyses of the acid digests and water samples was carried out by ICP-MS; (Agilent 8900 ICP-QQQ-MS) using (i) collision cell mode (He-gas) for Li, Be, B, Na, Mg, Al, P, S, K, Ca, Ti, V, Cr, Mn, Fe, Co, Ni, Cu, Zn, Ga, As, Rb, Sr, Y, Zr, Nb, Mo, Ag, Cd, Sn, Sb, Cs, Ba, La, Ce, Pr, Nd, Sm, Eu, Gd, Tb, Dy, Ho, Er, Tm, Yb, Lu, Hf, Ta, W, Tl, Pb, Bi, Th and U; and (ii) H_2_-reaction cell mode for Si (in plant material) and Se; and (iii) O_2_-reaction cell mode for As (at mass 91). Internal standards Sc, Ge, Rh, In, Te and Ir were employed to correct for signal drift^27^. For I analyses, the ICP-MS was operated in ‘no-gas’ mode and analysed separately as described in Watts *et al*.^24^, with all solutions analysed in a 0.5% TMAH matrix. For both analyses, an ISIS-3 sample introduction loop (Agilent Technologies, USA) was used to minimise the volume of sample (500 µL) presented to the ICP-MS to reduce the risk of carryover between samples.

Limits of detection are presented in Supplementary Tables (1, 3, 12) for soil, plants and water at the top of each column and analytical performance data are presented in full for certified reference materials at the bottom of each column for each determinand evidencing good analytical performance. The soil pH methodology utilised a 2 mm particle size and was based on a US EPA SW-846 Test Method 9045D for calcareous soils using 5 g of soil, stirred and mixed with a calcium chloride slurry (CaCl_2_) to a final ratio of 1:2.5. Organic matter content was determined by loss-on-ignition (LOI) at 450 °C for 1 g of soil, using a <53 µm particle size^75^.

### Influence of soil variables on plant micronutrient composition

Element concentration variables in both crop and soil were first natural log transformed due to their positively skewed distributions and to obtain approximately normal distributions. Associations between various soil parameters and micronutrient content of staple food groups were then investigated using multiple linear regression models. The relative importance of each predictor variable retained in models was also estimated, as the average over orderings variables, using the “relaimpo” R package by Groemping (2006)^76^. The relative contribution, seen as the fraction of total variance explained (R2), was reported as the percentage of the overall R2.

### Calculation of risk of deficiency

Food supply (g capita^−1^ day^−1^) data for each classified food item is summarised in Supplementary Table 5 for each country^66^, along with a correction for moisture content, showing the contribution of each food item to the overall dietary intake at a national level e.g. meat versus vegetables. For onward calculation of micronutrient supply, the daily food supply intake was multiplied by the MN concentration of the food item, as shown in Supplementary Tables 10 and 11 for Tanzania and Kenya, respectively, across three categories. These included for each food item, MN composition data originating from calcareous and non-calcareous soils in this study using food item data from Supplementary Tables 6 and 7 (food items) and 8 & 9 (food groups), compared to published MN data from FAO databases for Tanzania^9^ and Kenya^60^. The published MN composition data for both countries was not complete and therefore, supplemented with data from a comparable study in Malawi for I^24^ (Watts *et al*. 2015), Mo and Se^27^ – substituted data highlighted in the tables.

The calculation of the percentage risk of deficiency was calculated as described in Joy *et al*.^14,27^ and shown in Supplementary Tables 10 and 11. The sum of the dietary supply of MNs from all food items (mg capita^−1^ day^−1^) can be compared against published estimated average requirement (EAR) values for each micronutrient^77,78^ to provide an estimation of the percentage risk of deficiency. Where only recommended nutrient intakes (RNI) were available, conversion factors were applied to calculate the EAR^79^. For Zn and Fe, the EAR assigned for the lowest bioavailability of each was assumed.

## Supplementary information

Supplementary Dataset

## Data Availability

All supporting data is provided in supplementary tables to enable researchers to utilise the full dataset with metadata for other purposes.
